# Cyclodextrin-active natural compounds in food applications: a review of antibacterial activity

**DOI:** 10.3906/kim-2106-51

**Published:** 2021-09-14

**Authors:** Bing Ren TIAN, Yu Mei LIU

**Affiliations:** School of Chemical Engineering and Technology, Xinjiang University, Urumqi, China

**Keywords:** Cyclodextrin, active natural compounds, system, antibacterial activity, mechanism

## Abstract

Many natural compounds have excellent activity against different bacteria. However, their food use to inhibit the bacteria is often limited by poor water solubility, or instability to light, heat, oxygen, and other environmental factors. Cyclodextrin combines with these natural compounds could not only overcome these shortcomings, but also increase the antibacterial ability of active compounds. This review focuses on the following aspects of active natural compounds in cyclodextrin-based food: the preparation, food applications, and their possible antibacterial mechanisms of different systems. Both cyclodextrin and its derivatives are able to selectively combine with different guest molecules, such as terpenes, phenols and flavonoids, as well as essential oil and other plant extract. Finally, the opportunities and future challenges of active natural compounds in cyclodextrin-based food are outlined and proposed.

## 1. Introduction

In modern life, microbes could cause different levels of threat to food quality and even life safety, and even worse, result in zoonotic diseases [1–3]. Bacteria are closely linked to food storage [4–7]. In order to solve this problem, much progress has been made in food safety [8–10]. Developing food antiseptic products is usually requested to be safe, effective and environmentally friendly [11].

During the extraction and isolation of ingredients from plants, some natural organic molecules such as organic acids, essential oils and phenolic, are found to have good antibacterial activity against different bacteria/fungus [12–16]. However, these compounds with good antibacterial activity have inherent disadvantages such as poor water solubility, or instability against light, heat, and oxygen, which has been a significant limit for their application in the antibacterial field [17–20].

**Figure 1:**
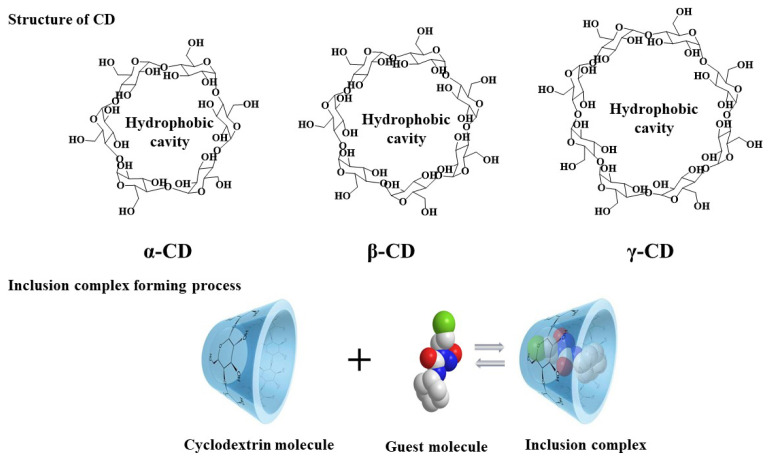
The structure of cyclodextrin and inclusion complex forming process.

Cyclodextrins (CDs), linked by α-1,4 glycosidic linkages, are a class of cyclic macromolecules formed by enzymatic hydrolysis of starch [21]. Natural CDs are divided into α, β, γ-CD, possessing appearance with internal hydrophobicity and external hydrophilicity [22] (Figure 1). Due to superb property, many hydrophobic organic molecules could form inclusion complexes with CD. These inclusion complexes can improve many chemical properties of guest molecules, such as enhancing solubility and stability, masking poor performance, and protecting from oxidative and photoinduced reactions [23]. Furthermore, when the CD forms into polymer, the range of application is improved in many fields, such as food [24,25], biocatalysis [26–28], and adsorption [29–32].

It is reported that the novel object combined CD with activated compounds (inclusion complexes, polymers) has superior performance in food antibacterial field [33–36]. In this review, we classify and compare different CD systems and their applications in food antibacterial field. First, the features of inclusion complexes and polymers are summarized and discussed. Moreover, the corresponding antibacterial applications and mechanisms are analyzed. In addition, future development of CD in antibacterial applications is proposed. Finally, the opportunities and challenges of different CD systems in antimicrobial applications have also been concerned.

## 2. Preparation of different systems of cyclodextrin-active naturals (CD-AN)

### 2.1. Inclusion complexes of CD-AN

The antimicrobial activities of many hydrophobic organic molecules have been experimentally determined [37,38]. Natural CDs have a definite solubility in water because of existing hydrophilic side chains [39]. In order to improve the various properties of the active naturals, many methods of preparing CDs inclusion complexes with activate naturals, have been adopted, including in saturated aqueous solution (SAS) [40,41], under solid phase conditions (milling) (SPC) [42], under heterogeneous conditions, liquid-liquid process [43,44], ultrasonication [45], freeze-drying [46], and spray drying [47]. Preparing CD inclusion complexes in aqueous solutions is appropriate for hydrophobic guest molecules, such as linoleic acid [48]. Preparing CD inclusion complexes under solid phase conditions is more suitable for some volatile molecules [49]. Recently, ultrasound has attracted more and more attention due to its advantages of simplicity, energy saving, high efficiency, and environmental protection [50,51]. Therefore, ultrasound is not only broadly involved in various food industries, including preservation, processing, and extracting processes [52], but also popular in preparation of inclusion complex [53].

### 2.2. Polymers of CD-AN

Although the inclusion complexes have many applications, their shortcomings are worth considering. For example, some CD inclusion complexes exhibit instability under acid-base conditions, and it is easily affected by the size of the guest molecule to preparation. In the current research, the polymers of CD with activated naturals have become much more popular than ever. As the synthesizing concept of polymer was introduced into CDs, it is depicted that the antibacterial activity might be enhanced when a small molecule possessing antibacterial activity was supported on CD polymers [54,55]. CD polymers also have a broad range of applications in the preparation of antibacterial materials [56,57]. The methods of preparation for CD polymers have been developed, such as reversible addition-fragmentation transfer polymerization (RAFTP), ring opening polymerization (ROP), and atom transfer radical polymerization (ATRP) [56]. However, some defects have been observed in preparing polymers. For example, it will consume large energy during the preparation process.

## 3. Applications of CD-AN

### 3.1. Inclusion complex of CD-AN

Many natural extracts have good antibacterial effects, such as terpenes, phenols, and flavonoids. However, many of the substances show instability to factors such as temperature, oxygen, light, and other factors, as well as poor water solubility. After the formation of the corresponding CD inclusion complexes, some properties of active natural compounds will be improved such as antibacterial activity, water solubility, stability and so on. In addition, medicinal plant extract often can be utilized alone, additively or synergistically to improve the therapeutic efficiency of other drugs, and thus serves as prototype molecules for pharmacological research [58].

**Figure 2:**
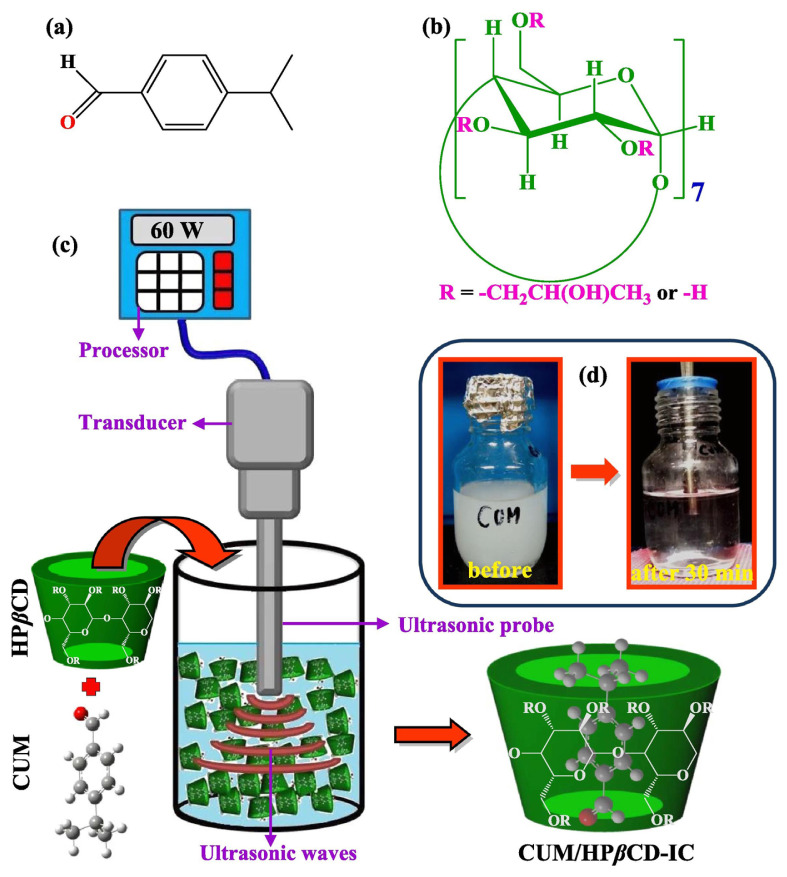
Chemical structure of (a) cuminaldehyde (CUM), (b) 2-hydroxypropyl-β-cyclodextrin (HP-β-CD), (c) schematic representation of ultrasound processed inclusion complex (IC) formation between HP-β-CD and CUM, and (d) photographs of CUM/HP-β-CD-IC solution before and after ultrasonication (30 min). Copyright 2019. Reproduced from the Elsevier.

α-Bisabolol, known as levomenol, is a sesquiterpene monocyclic alcoholic found in many medicinal plants. A number of studies have reported that α-bisabolol has multiple effects, including antiinfective, antioxidant and healing properties, and inhibition of mast cell sensitization [59,60]. In order to unfreeze the limitation of solubility of α-bisabolol, pharmaceutical and cosmetic industries are increasingly using drug carrier systems with CDs to enhance the physicochemical and pharmacological properties of hydrophobic drugs while also seeking to reduce their side effects [60]. The inclusion complex was prepared by β-CD and α-bisabolol, and the minimum inhibitory concentration (MIC) was determined by broth microdilution technique using *S. aureus*, *E. coli*, and *P. aeruginosa* as strains. The results revealed the independent β-CD was in direct contact with gram-negative or gram-positive bacteria and did not exhibit antibacterial ability, while the inclusion complex combined of α-bisabolol with β-CD had a direct antibacterial effect on *S. aureus* [60]. Another similar example is the cumylaldehyde which is the main essential oil of cumin seed. Cui et al. [51] applied ultrasound to prepare cumin essential oil with CD to form inclusion complexes. By comparing the control group, the experimental group decreased in the surviving population of both gram-negative and gram-positive bacteria (Figure 2). In addition, monoterpenes have antibacterial properties, but their strong odors and wrong taste frequently influence their use in food. In order to improve this phenomenon, the thymol and linalool were prepared as inclusion complexes with CD. The similar experiment results indicated an obvious difference between the inclusion complex and the monomer in the antibacterial ability test for *E. coli* and *S. aureus* (p < 0.05). These monoterpenes after forming inclusion complexes exhibited antimicrobial activity against *S. aureus* was 1.4–3.4 times more effective than their free state. Furthermore, it has been found that the inclusion complexes could achieve the same level of inhibition efficiency as the monomers at lower concentrations [61]. Other experiments have found that the combination of natural products (d-limonene) and synthetic antibiotics (gentamicin) could improve the antibacterial ability on the basis of the original [62]. This finding has led researchers to be more interested in exploring natural antibacterial antibiotics.

Over the years, antibiotics have led to the development of microbial resistance [63,64]. Therefore, it is difficult to select effectively antibiotics. Many scientists have long been keen to look for plant active ingredients as the substitution of antibiotic to fight against pathogenic microorganisms. Research results indicated that natural antibacterial compounds combined with antibiotics could be able to improve the antibacterial efficiency [62,65]. For example, d-limonene discovered synergism against *S. aureus* 10 with MIC reduction from 13.71 μg/mL to 4 μg/mL as associated with gentamicin, and against *E. coli* 06, where the d-limonene association with gentamicin was able to reduce the MIC from 30 μg/mL to 20.1 μg/mL [62]. Caffeic acid, derived from hydroxycinnamic acid, has antibacterial and antioxidant biological properties [66]. The sensitivity of the carboxyl group of caffeic acid to severe environments (pH range: 4.5–7) can affect its antibacterial activity, as well as its sensibility to oxidation. Besides, it was discovered that caffeic acid formed inclusion complex with different CD could enhance the antibacterial activity [67]. Though natural active compounds (caffeic acid, ellagic acid, quercetin, and polyphenolic compounds) have much lower antibacterial ability than chemically synthesized antibiotics (ciprofloxacin, isoniazid) [68], the dosage can be increased to achieve the purpose of treatment in practical application.

Polyphenolic compounds are also ubiquitous in plants. Since the successful separation from plants, many phenolic compounds have been proved to possess strong antibacterial activity [69]. Resveratrol is a natural polyphenol with several biological activities such as antioxidation, antibacterial, and antiinflammatory [70,71]. Its application gets stuck due to its low water solubility. After resveratrol formed inclusion complexes with CD, the results displayed that the corresponding inclusion complex was more water-soluble than the monomer. Similarly, in the antibacterial experiments on *C. jejuni* and *C. coli*, it was discovered that the inclusion complex had significant antibacterial effect compared to resveratrol monomer [72–74]. Furthermore, concerned about the toxicity of synthetic compounds, the consumers are inclined to choose the natural organic ingredients which has low toxicity [75–78]. Carvacrol is a phenolic compound adopted by the FDA for use as a food additive [79]. Correlated studies have reported that carvacrol has antiseptic effects in the pharmaceutical, agricultural, cosmetics and food industries [80]. After introducing CD, the experimental results revealed that the CD inclusion complex did not enhance the restraint rate of *E. coli* K12 and *S. Typhimurium* compared to without inclusion [81,82]. Given this reason, it could be found that after formation of the clathrate, the CD “masks” part of the structure of the original compound, so that the activity of the compound is reduced or disappeared. The solubility of inclusion complex in water was greatly improved, which will be helpful to promote its industrial applications by increasing the amount of inclusion complexes to achieve the expected antibacterial effect [83]. Hesperidin is a flavonoid glycoside found in citrus peel [84]. The complexation of the insoluble compound with the CD improves its water solubility, the stability of light and oxygen or odor removal, thus enhancing its biology without changing its original structure. Corciova et al. [85] successfully encapsulated hesperidin by using CD, and the antibacterial activity of *S. aureus* ATCC 25923, *E. coli* ATCC 25922 and *C. albicans* ATCC 10231 were evaluated by agar diffusion method. The results revealed that the prepared inclusion complexes had higher antibacterial activity than hesperidin.

Apart from active monomers, many natural plant extracts have been widely applied for many years in daily life [86]. Studies have evaluated that many plants have essential biologically active compounds such as mixed essential oils [87–89]. Black pepper is typically applied in medicine, diet, preservation, and biological preparations because of containing black piperine [90,91]. Related experiment has corroborated that quality of black pepper oils are sensitive to environmental factors [92]. Therefore, CD was chosen as the “coat” to improve the stability of essential oils. Different functional groups of CD would affect antioxidant activity of essential oil, but it was reported in the antibacterial experiment that the activity against *S. aureus* and *E. coli* was increased while forming inclusion complexes [81,93,94]. The active ingredient terpenoids in rosemary essential oils also have a good effect on a variety of bacteria and fungi [95]. Even though rosemary essential oils acted as flavoring agents and antibacterial agents in food, their thermal stability restrains the range of application in processed food. However, when essential oils were encapsulated by CD, it was discovered that the thermal stability was considerably improved and maintained the original antibacterial ability of essential oil [96]. Guava is a traditional medicine for a long time based on its bioactivities. The Guava leaf essential oils are mainly composed of limonene, β-caryophyllene, 1,8-cineole, and α-pinene, which have antiproliferative, antioxidative, and antibacterial effects [97]. Encapsulating essential oils with CDs improves their water solubility and activities in food. In the corresponding antibacterial experiments, it was discovered that the antioxidant activity of the inclusion complexes was 26%–38% stronger than those without being encapsulated. Meanwhile, the antimicrobial activity increased 4 and 2 times against *S. aureus* and *E. coli*, respectively [98].

Due to peculiar properties, volatile oil has become an important antibacterial additive in food because they meet consumer demand for foods that do not contain synthetic chemical preservatives. Related studies have reported that garlic oil, controlling microbial growth, is one of the most commonly utilized essential oils [99]. Many researchers discovered that garlic oil has distinct antibacterial properties in various concentrations [100] and several strains such as *E. coli* [101,102], *L. monocytogenes*, *S. enteritidis*, and *S. aureus* [103]. However, garlic oil is also easily affected by corrosive substances. Therefore, the inclusion with CD was applied to increase the stability of garlic oil and to expand the antibacterial effect. The experimental results showed that the inclusion compound exhibited a good antibacterial effect against *S. aureus*. Moreover, in order to inhibit *E. coli*, the garlic oil/β-CD complexes with and without heat treatment experiment indicated that they displayed inhibitory effects at the experiment conditions (60 °C) [104]. Antibacterial effects of inclusion complex of CD-AN are shown in Table 1.

### 3.2. Polymers of CD-AN

Although CD has a certain degree of application in forming inclusion complexes with natural compounds, in order to expand the application of natural antibacterial substances, researchers have applied CD to generate polymers. The polymers exhibit better stability than the inclusion complexes and take less preparation time than the inclusion complexes. Therefore, researchers will choose the preparation method according to different needs. Corresponding experimental results indicated that the polymers composed of CD and active natural product have a certain improvement in the corresponding antibacterial properties [107].

Thymol is an antibacterial and antioxidant monoterpene compound. However, its application is restricted by its hydrophobicity and volatility. The polymer of CD with thymol has been successfully applied to the pork preservation system to prevent oxidation and prolong the storage time of the meat at a relative humidity of 75% [108]. The corresponding antibacterial experiments showed that the antibacterial ability of the polymer added with CD into material was better than that of the polymer without CD added.

A new nanofiber web is prepared by electrospinning using CD and limonene/quercetin/a-tocopherol/eugenol/carvacrol. It has been demonstrated that the obtained nanofiber web has high antibacterial activity against *E. coli* and *S. aureus*, and it would be widely used in the fields of food and oral care by its well thermal stability and quality antibacterial ability [109–113]. Polyvinyl alcohol, cinnamon essential oil, and CD nanofibers prepared by electrospinning techniques have been also successfully prepared under optimal processing conditions. The bacteria were inhibited by the sustained release of the loaded cinnamon essential oil in the system. The cross-linked nanofiber membrane had good in vitro antibacterial properties against *S. aureus* and *E. coli*, such as prolonging the storage time of the mushroom [114].

A new material that can be investigated in food controlled release packaging systems has been successfully developed and characterized by Saini et al. The CD was directly grafted onto the carboxyl group of the temperature-oxidized cellulose nanofiber (TEMPO-CNF), and then the aromatic essential oil component of carvacrol was embedded in the TEMPO-CNF of the grafted CD. In the antibacterial activity test for *Bacillus subtilis*, it was found that the antibacterial time of the new material was extended to 50 h from 3 h (or increased 47 h). These promising results paved the way for the development of novel bio-based controlled release packaging materials with high antimicrobial activity [115]. In addition, a new carotenol-loaded CD cellulose packaging material was also reported, in which the continued release of carvacrol in the material was considered to be up to 21 h in the relevant antibacterial experiments. Therefore, the material can be served as a new type of bio-based food packaging material, and the food can be better preserved and prolonged the shelf life by the sustained release of the antibacterial molecule [116].

After the CD is prepared into polymers or nanoparticles by different methods, some compounds, such as cinnamon essential oil, are often added to these newly synthesized materials to impart better antibacterial effects [54,117]. Related studies also have exhibited that linalool has antibacterial, antiinflammatory, local anesthetic, analgesic and antitumor effects [118]. In order to overcome the disadvantages of linalool in daily application, it has been studied to form inclusion complexes with CD to increase its use in food and medicines. Recently, Aytac et al. [119] explored different kinds of CD to form polymers by electrospinning techniques. It is worth noting that the corresponding polymer showed rapid solubility in water (2 s). In addition, in the antibacterial experiment, it was discovered that different kinds of polymers had potent antibacterial activity against *E. coli* and *S. aureus* by using the living cell counting method.

**Figure 3:**
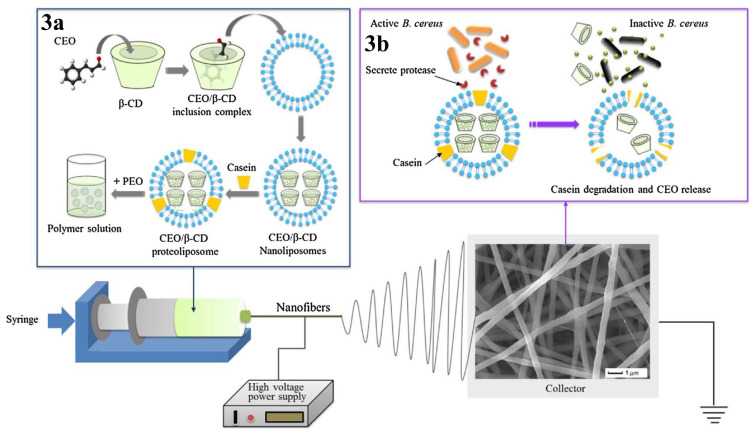
(3a) Schematic of electrospinning for cinnamon essential oil/β-cyclodextrin (CEO/β-CD) proteoliposomes incorporated into poly(ethylene oxide) (PEO) nanofibers. (3b) Schematic of *Bacillus cereus* (*B. cereus*) proteinase-triggered CEO release from CEO/β-CD proteoliposomes. Copyright 2017. Reproduced from the Elsevier.

During food storing, it is necessary to improve the packaging materials on the outside. A nanomaterial was successfully produced by electrospinning technology from cinnamon essential oil (CEO), CD proteoliposomes, and polyethylene oxide (PEO). The nanomaterial was reported to have no effect on the sensory quality of beef during experimental applications [120] (Figure 3).

In addition, Cui et al. [121] also studied the antibacterial and antiseptic effects of nano-material film formed with tea tree oil, CD, and polyethylene oxide. The experimental results indicated that the antibacterial activity of the nanomaterial film was obviously enhanced after plasma treatment, which can prolong the shelf life of beef. When the CD was prepared as a nanomaterial and loaded with the essential oil, the polymer revealed inhibition to the growth of all experimental bacteria, which indicated that this type of polymer could be a viable candidate for use in antimicrobial packaging materials in the food industry [122]. The results of polymer of CD-AN are displayed in Table 2.

## 4. Mechanism of CD-AN

The inclusion complexes/polymers clearly show their good antibacterial effect from a macroscopic point of view. In a more in-depth study, the action mechanism of inclusion complexes/polymers can be summarized as follows:

Firstly, hydrophobic molecules interact with the cell membrane, causing cell membrane rupture. Some molecules, including oregano/dill/cilantro/coriander/eucalyptus essential oils, thymol, carvacrol, cinnamaldehyde, linalool, have antibacterial activity due to their hydrophobicity and free hydroxyl groups [123,124]. Due to their hydrophobicity, small molecules interact with the cell membrane lipid bilayer, aligning themselves between fatty acid chains, resulting in swelling and instability of the membrane structure, increasing its fluidity and permeability. In addition, the presence of free hydroxyl groups and delocalized electronic systems is also important. As a proton exchanger, the gradient on the cell membrane is reduced, and eventually the cell death occurs due to a decrease in ATP (adenosine triphosphate, which transfers the chemical energy in the cell) [116,125–127]. A case in point is that the terpene component in the essential oil could destroy the cell membrane of the bacteria, so the essential oil has antibacterial activity [128]. However, the antimicrobial activity of some essential oil components against *S. aureus* differs from that of *E. coli*, which may be related to the cell wall composition variance between gram-negative and -positive bacteria. Since *E. coli* have a thin layer of peptidoglycan and an outer layer composed of lipoprotein, lipopolysaccharide and phospholipid, while the cell wall of *S. aureus* consists of a layer of peptidoglycan with many pores and has a porous cell wall structure. Some essential oils, due to their lipophilic characteristics, lead to an increase in membrane fluidity and permeability, and loss of function within the gram-positive bacteria; others can enter the gram-negative bacteria through the pores and lead to bacterial death [108,119,129]. In addition, many researchers have found that some active ingredients (resveratrol) can be divided into four steps: the diffusion of the complex in solution; the collision with the bacterial membrane; the dissociation and interaction of the complex of the guest molecule with the bacterial membrane [72,73].

Secondly, some studies have found that antibacterial active sites are present in the membrane or cytoplasm of bacteria, increasing the permeability of the antimicrobial agent so as to kill bacteria. For example, the original site of antibacterial activity (eugenol, black pepper essential oil) displayed in cell membranes and cytoplasm. By increasing the water solubility of guest molecule (such as essential oils), CD can improve the infiltration of guest molecule into these areas to kill bacteria [93,130]

Thirdly, CDs could increase the solubility of antimicrobial molecules, allowing more active molecules to interact with bacteria. The antibacterial activity of some molecules appears to be concerned with the ability of the molecule to interact with electrons on the bacterial surface or in the cytoplasm. This may be due to the increased solubility of small molecules, reduced formation of agglomerates, or small molecules with high activity interact with bacteria, which might be caused by CD [74,131].

## 5. Comparation with recent study

**Figure 4:**
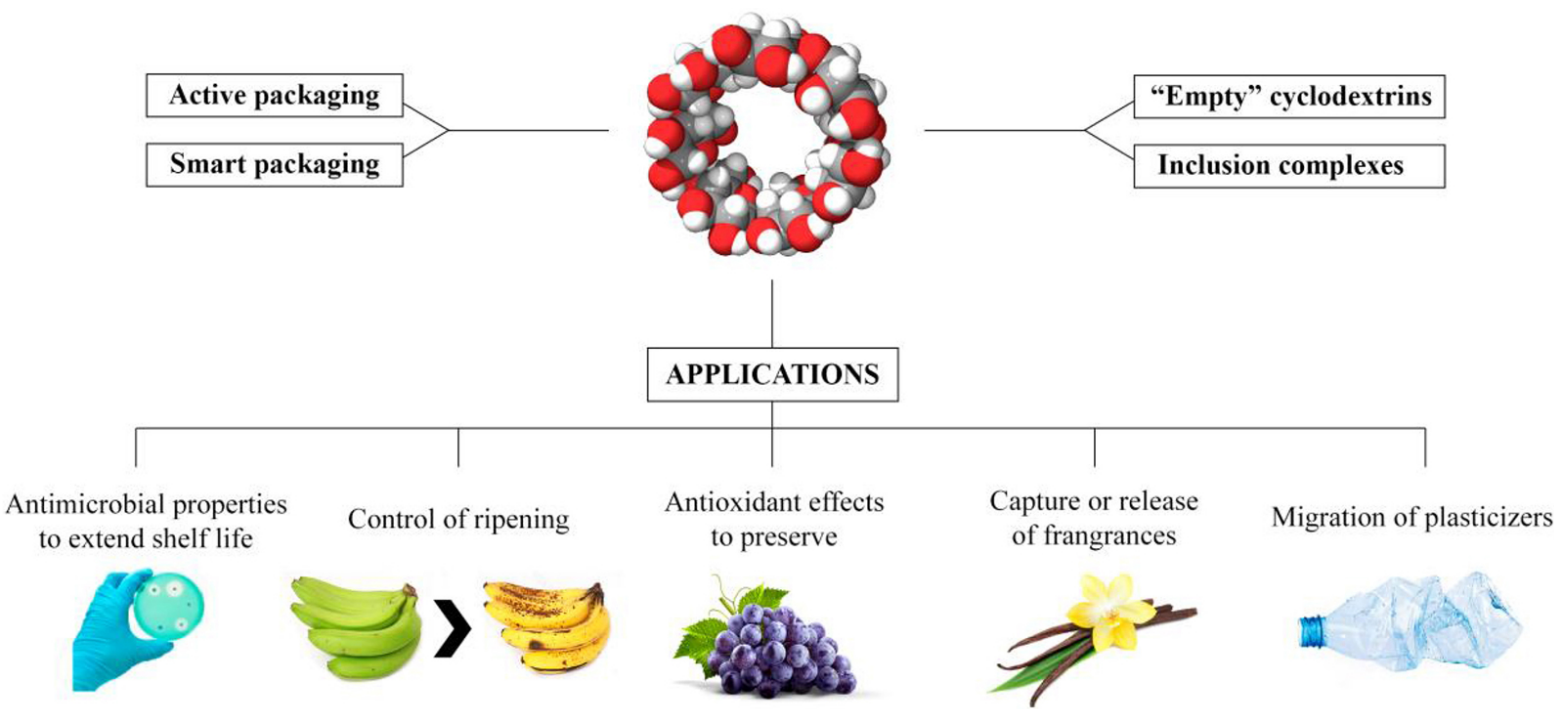
Graphical representation of the applications of CDs in food packaging. Copyright 2020. Reproduced from the Elsevier.

The application of CD-AN in the food industry has been fully demonstrated in recent studies [132–136] (Figure 4). The characteristics of CD (physical chemistry, toxicity, etc.), the regulations and laws governing the use of CDs in the food industry, and the general trend of more widespread acceptance of CDs as a food additive all received extensive attention, including the latest development of CDs as a carrier and its antibacterial mechanism, as well as application in the food industry. Recently, for CD loaded with different active ingredients for various foods (vegetables, meat, carbohydrates and starchy foods), this article also summarizes and compares the latest developments (Table 3). This analysis discovered that although a large number of active compounds have been synthesized by researchers, there are few reports on their applications. Therefore, the characteristics of active compounds should be combined to discover their specific application value, and basic research should create value in practice.

## 6. Conclusion and future prospect

Many natural active substances with significant antibacterial activity have been employed, and they are promising natural antibacterial drugs. These active substances have been prepared into inclusion complexes and polymers by the aid of CDs, and corresponding experimental verifications have been performed demonstrating successful antibacterial results. Although CDs were often utilized to enhance solubility and stability of some compounds, the CD inclusion complexes did not always effectively increase the antibacterial activity of these compounds. Some studies displayed that CD inclusion complexes could enhance the antibacterial activity when they are combined with some antibiotics. For example, d-limonene that revealed relevant clinical antibacterial activity for both gram-positive and gram-negative bacteria as well as a synergistic effect when associated with gentamicin [62]. Researchers found that the parent of CD showed no antibacterial activity, but the CD combine with α-bisabolol could modulate the antibacterial action of CD [60]. It could be indicated that complexation has altered physicochemical interaction with the cellular system, including the compound/antibiotic interaction. In future related research, it is necessary to conduct more in-depth exploration of the relevant details.

Based on the above research, we can speculate on the future application prospects of CD-AN. The corresponding outlook is as follows: (1) Because of excellent property of antibacterial performance and safety, the inclusion complexes of CD-AN can be applied in a variety of food systems to minimize the risk, including thermal processing, storage with high temperatures, transportation and sale. In addition, it is necessary to explore the bioactivities of the compounds regarding their antibacterial and antibiotic modulatory activity by means of the experiment results. (2) In the aspect of polymer (CD-AN), we notice that some novel antibacterial packaging have broad prospect in the field of meat production preservation. And some nanofibrous webs has excellent properties, high preservation time, and slow release; therefore, it may be used as fast-dissolving supplement material in food, and biomedical products.

The FDA has approved CD and its derivatives for use in humans at safe concentrations. In addition, there are currently about 20 synthetic drugs for CD coordination strategies on the market, which aims for the treatment of microorganisms, cancer, central nervous system diseases, and cardiovascular system diseases. CD inclusions should be considered an important step in the design of new phytochemicals for herbal or health supplements. Phytochemicals are inexhaustible wealth on earth. It is necessary to further study their interaction with CDs including types and factors affecting interactions, complex preparation strategies, and developed complexes, the characterization and biological evaluation of in vitro and in vivo models. In this way, we can expect CD and its derivatives to help deliver phytochemicals at target sites, which may solve the toxicity problems associated with synthetic drugs. What is more, in the subsequent development, the CD nanomaterial polymers also demonstrate an upward trend, and they have broad application prospects in the future due to their advantages that many CD inclusion complexes do not possess. Although the exact antibacterial mechanism of antibacterial active substances has not been explained clearly, the following main mechanisms have been found, such as destroying the bacterial cell wall, increasing the water solubility of the active substance, and acting on the internal system of the active molecule. The specific role of CD in the antibacterial system is also currently available which indicates that CD mainly increases the solubility of active molecules and causes bacterial rupture to die. Future research should focus on the preparation of new antibacterial materials, the discovery of functional molecules, and their antibacterial mechanisms. In addition, reducing the cost of material preparation is an urgent issue to be solved. Moreover, in the current research, it is limited to study the activity of the inclusion complex and the polymer, respectively. Therefore, they should be compared to each other so as to find the advantages and disadvantages in the same system in future research when applied to different scopes. Therefore, the application of CD-AN in food will be more extensive with the development of technical means.

## Figures and Tables

**Table 1:** Application of CD-AN (inclusion complexes) in antibacterial effect.

Active naturals	Bacterial species	Type of CD	Methods	Results	References
Cuminaldehyde	*E. coli*; *S. aureus*	HP-β-CD	SAS	The surviving population of both tested bacteria decreased by 100%, compared to the control group after inclusion, and the water solubility was also enhanced.	[51]
Thymol	*E. coli* CECT 943; *S. aureus* CECT239	HP-β-CD	SAS	The solid complexes of thymol showed higher antimicrobial activity for both *E. coli* (3.4 times) and *S. aureus* (2.2 times). The inclusion complexes had the promising future in nutritional or therapeutic applications.	[61]
Caffeic acid	*S. epidermidis* ATCC 12228; *S. aureus* ATCC 6538; *K. pneumoniae* ATCC 11296	β-CD/HP-β-CD/M-β-CD	SAS	While the inclusion complexes solution, maintaining 24 h at 25 °C and 50 rpm on dark, was placed in ultrasounds bath during 30 min, it was found that M-β-CD could not form a stable inclusion complex the change in pH (pH = 3 and 5) had little effect on the inclusion ratio and antibacterial effect of β-CD/HP-β-CD.	[67]
Linalool	*E. coli* CECT 943; *S. aureus* CECT 239	HP-β-CD	SAS	The results showed higher antimicrobial activity for both *E. coli* (69% growth inhibition at a concentration of 3.53 mM) and *S. aureus* (78% growth inhibition at a concentration of 12.92 mM) after 24 h of incubation.	[61]
d-Limonene	*E. coli* 06/ATCC 25922; *S. aureus* 10/ATCC 6538; *P. aeruginosa* 24/ATCC 9027	β-CD	SPC	When inclusion complexes associated are shared with gentamicin/norfloxacin, they are found to have a synergistic antibacterial effect on MIC (*S. aureus* (0.4 μg/mL); *E. coli* (20.1 μg/mL); *P. aeruginos*a (32 μg/mL). However, further research was still needed on the mechanism to explain the explanation.	[62]
α-Bisabolol	*E. coli*; *P. aeruginosa; S. aure*us	β-CD	SPC	The results showed that the MIC (*S. aureus*) of the inclusion complex (406.37 μg/mL) was lower than that of the monomeric compound (161.27 μg/mL). However, when the inclusion complexes associated with gentamicin/norfloxacin, they were found to have a synergistic antibacterial effect.	[60]
Carvacrol	*E. coli* K12/CECT 943; *S. aureus* CECT 239; *S. Typhimurium*	β-CD	SAS/SPC	There was an improvement in inhibition of 65% (SAS: 300 μg/mL) and 70% (SPC: 350 μg/mL) for *E. coli* and 68% (SAS: 300 μg/mL) and 72.7% (SPC: 350 μg/mL) for *S. Typhimuri*um, respectively. Moreover, the stability of guest was greatly improved.	[83]
		HP-β-CD	SAS	The MIC was achieved for SAS for both microorganisms (2.44 mM in *E. coli* and 2.61 mM for *S. aureus*), and the complexes required (14.60 mM) to achieve total growth inhibition against *E. coli*.	[61]
trans-Resveratrol	*A. butzleri*; *C. coli* 219872/53/873/ATCC 33559; *C. jejuni* 225421/ATCC 33560	M-β-CD	SAS	The aqueous dissolution of complexation increased 400 times, compared to the original. Furthermore, the results showed good antibacterial activity against *Campylobacte*r spp. with MIC values ranging from 64 to 512 μg/mL.	[73]
		HP-γ-CD	SAS	The results showed good antibacterial activity against different bacteria with MIC values ranging from 64 to 256 μg/mL.	[72]
Hexahydro-β-acids	*L. monocytogenes*	M-β-CD	SPC	The water solubility increased remarkably (up to 0.83 mg/mL). Particularly, the inhibition diameter of the complex against *L. monocytogenes* at different concentrations (300, 200, 100 μg/mL) were up to 14.2 ± 0.5, 12.5 ± 0.8, and 9.3 ± 1.1 mm, respectively.	[105]
Hesperidin	*E. coli* ATCC25922/ATCC 25922; *S. aureus* ATCC 25923; *C. albicans* ATCC 10231	β-CD	SPC/SAS	In in vitro study, the inclusion complexes by different methods had enhanced the antibacterial activity (IC_50_) (SPC: 0.0484 ± 0.69 mM) (SAS: 0.0422±0.74 mM), compared to the free compound (0.0565 ± 0.80 mM).	[85]
Black pepper oil	*E. coli; S. aureus*	HP-β-CD	SAS	The antibacterial activity of black pepper oil was improved by 4 times against both *S. aureus* and *E. coli*; and the stability was increased.	[93]
Garlic oil	*E. coli*; *S. aureus*	β-CD	SAS	The formed inclusion complexes could not only protect garlic oil at the thermal treatment twice as high as the volatilization temperature (30°C) of the free, but also attain the best antibacterial effect at 81.73 mmol/L of garlic oil.	[104]
Guava leaf oil	*E. coli*; *S. aureus*	HP-β-CD	SAS	Encapsulation increased the antibacterial activity against both *S. aureus* and *E. coli* by 4 and 2 times, respectively.	[98]
Rosemary essential oil	*C. tropicalis*; *L. monocytogenes*; *S. pastorianus*; *S. Typhimurium*	β-CD	SAS	The β-CD could protect the guest to affect by high temperatures (75 °C) and maintain its antibacterial activity compared to untreated oil. The minimal inhibitory concentrations (MIC) of the inclusion complexes for *S. Typhimurium*, *L. monocytogenes*, *C. tropicalis*, and *S. pastorianus* were 14.66, 14.14, 2.05, and 3.07 mg/mL, respectively.	[96]
Hyptis martiusii Benth Essential oil	*S. aureus* ATCC 25923; *P. aeruginosa* 15/ATCC 9027; *E. coli* 06/ATCC 25922	β-CD	SPC	While forming inclusion complexes, it was found that the complexes had no antibacterial action. However, when the inclusion compound was combined with gentamicin, it had antistaphylococcus activity and synergistic antibacterial action against gram-negative bacteria.	[106]

**Table 2:** Application of CD-AN (polymer) in antibacterial effect.

Active naturals	Bacterial species	Type of CD	Methods	Results	References
Linalool	*E. coli* ATCC 10536; *S. aureus* ATCC 25923	M-β-CD/HP-β-CD/HP-γ-CD	ELS	The antibacterial activity of polymers of HP-β-CD (69%), M-β-CD (65%), and HP-γ-CD (45%) was about 84%, 93%, 95% against *E. coli*, and 70%, 79%, and 88% against *S. aureus*, respectively. In addition, the water solubility of guest molecule was increased.	[119]
Thymol	*E. coli* ATCC10536; *S. aureus* ATCC 25923	γ-CD	ELS	The growth inhabitation rate of inclusion complexes was 76.4 % (1:1), and 85.0% (1:2) against *E. coli*, and 85.2%, and 86.6 % (1:2) against *S. aureus*, respectively.	[110]
Limonen	*E. coli* ATCC10536; *S. aureus* ATCC 25923	M-β-CD/HP-β-CD/HP-γ-CD	ELS	The polymer with M-β-CD released much more limonene at 37, 50, and 75 °C than HP-β-CD and HP-γ-CD ones, and an inhibitory rate of about 77%, 79%, 93%, and 90% against *E. coli* and about 70%, 96%, 97%, and 85% against *S. aureus* for free limonene, HP-β-CD M-β-CD, and HP-γ-CD, respectively.	[109]
Cinnamon essential oil	*B. cereus* ATCC 14579	β-CD	ELS	Compared with the control group (the preserving beef sample without polymers), the reduction in population of, 99.6%. 99.9%, 99.99%, and 99.999% was observed at 4 °C, 12 °C, 25 °C, and 37 °C after 4 days, respectively. The results showed that the antibacterial efficiency of polymer against *B. cereus* was positively associated with temperature.	[120]
Tea tree oil	*E. coli* O157:H7	β-CD	Coprecipitation method	After preserving the beef for 7 days, the inhibition efficiently of 99.99% was observed whether at 4 °C or 12 °C. In addition, the encapsulation efficiency of polymer could reach 73.23% at 60 °C.	[121]
Coriander essential oil	*B. thermosphacta* CECT 847; *C. coli* 22/08/ATCC 33559; *C. jejuni* 930/12/ATCC 33560; *E. coli* O157:H7; *L. monocytogenes* CECT 911; *Y. Enterocolitica* CECT 500	α-CD/β-CD/HP-β-CD	Carbonyldiimidazole (cross-linking agent)	The dissolution studies indicated that dissolution in acetone was faster and resulted in an almost complete dissolution of the oil major compounds previously incorporated. In addition, the new materials were stable at temperatures over 200 °C.	[122]

**Table 3:** Recent study of CD-AN applied in different food.

CD-AN	Bacterial species	Suitable food	Reference
Cuminaldehyde/β-CD	*E. coli* O157:H7	Vegetable juices	[137]
Tea tree oil/hydroxypropyl-β-CD	*M. fructicola*	Peach fruit	[138]
p-Anisaldehyde/β-CD	*R. stolonifer*; *A. niger*; *Penicillium*	Strawberry	[139]
Essential oils/β-CD	-	Green/red and yellow peppers	[140]
Essential oils/β-CD	-	Tomato	[141]
Ferulic acid/β-CD	*P. fluorescens*; *S. putrefaciens*; *S. aureus*; *E. coli*; *S. typhimurium*; V. anguillarum	Hairtail	[142]
Cinnamaldehyde/β-CD	*E. coli*; *S. aureus*	Cold fresh pork	[143]
Oregano essential oil/β-CD	*E. coli*; *L. monocytogenes*	Purple Yam	[144]
Egg white protein/β-CD	-	Silver carp	[145]
Geranyl acetone/β-CD	-	Cigarette flavoring	[146]
Quercetin/β-CD; Quercetin/γ-CD	-	Fresh cheese	[147]
Orange essential oil/β-CD	*A. terreus*; *A. niger*	Bakery products	[148]

## Abbrevitaions

CD: cyclodextrin
CD-AN: cyclodextrin-active naturals
CEO: cinnamon essential oil
CUM: cuminaldehyde
β-CD: β-cyclodextrin
HP-β-CD: hydroxypropyl-β-cyclodextrin
M-β-CD: methylated-β-cyclodextrin
γ-CD: γ-cyclodextrin
SAS: saturated aqueous solution
SPC: solid phase conditions (milling)
ELS: electrospinning
MIC: minimum inhibitory concentration
